# Esterification of Alginate with Alkyl Bromides of Different Carbon Chain Lengths via the Bimolecular Nucleophilic Substitution Reaction: Synthesis, Characterization, and Controlled Release Performance

**DOI:** 10.3390/polym13193351

**Published:** 2021-09-30

**Authors:** Xiuqiong Chen, Qingmei Zhu, Chang Liu, Dongze Li, Huiqiong Yan, Qiang Lin

**Affiliations:** 1 Key Laboratory of Tropical Medicinal Resource Chemistry of Ministry of Education, College of Chemistry and Chemical Engineering, Hainan Normal University, Haikou 571158, China; chenxiuqiongedu@163.com (X.C.); zhuqm03@163.com (Q.Z.); 2 Key Laboratory of Natural Polymer Functional Material of Haikou City, College of Chemistry and Chemical Engineering, Hainan Normal University, Haikou 571158, China; liuchang202107@126.com (C.L.); dongzeli2019@163.com (D.L.); 3 Key Laboratory of Water Pollution Treatment & Resource Reuse of Hainan Province, College of Chemistry and Chemical Engineering, Hainan Normal University, Haikou 571158, China

**Keywords:** alkyl alginate ester derivative, bimolecular nucleophilic substitution reaction, controlled release performance, cytocompatibility, hydrophobic pharmaceutical formulations

## Abstract

To extend the alginate applicability for the sustained release of hydrophobic medicine in drug delivery systems, the alkyl alginate ester derivative (AAD), including hexyl alginate ester derivative (HAD), octyl alginate ester derivative (OAD), decyl alginate ester derivative (DAD), and lauryl alginate ester derivative (LAD), were synthesized using the alkyl bromides with different lengths of carbon chain as the hydrophobic modifiers under homogeneous conditions via the bimolecular nucleophilic substitution (S_N_2) reaction. Experimental results revealed that the successful grafting of the hydrophobic alkyl groups onto the alginate molecular backbone via the S_N_2 reaction had weakened and destroyed the intramolecular hydrogen bonds, thus enhancing the molecular flexibility of the alginate, which endowed the AAD with a good amphiphilic property and a critical aggregation concentration (CAC) of 0.48~0.0068 g/L. Therefore, the resultant AAD could form stable spherical self-aggregated micelles with the average hydrodynamic diameter of 285.3~180.5 nm and zeta potential at approximately −44.8~−34.4 mV due to the intra or intermolecular hydrophobic associations. With the increase of the carbon chain length of the hydrophobic side groups, the AAD was more prone to self-aggregation, and therefore was able to achieve the loading and sustained release of hydrophobic ibuprofen. Additionally, the swelling and degradation of AAD microcapsules and the diffusion of the loaded drug jointly controlled the release rate of ibuprofen. Meanwhile, the AAD also displayed low cytotoxicity to the murine macrophage RAW264.7 cells. Thanks to the good amphiphilic property, colloidal interface activity, hydrophobic drug-loading performance, and cytocompatibility, the synthesized AAD exhibited a great potential for the development of hydrophobic pharmaceutical formulations.

## 1. Introduction

In recent years, synthetic amphiphilic polymers have drawn more and more attention from researchers, and their self-aggregation performance has also become a research hotspots [1,2], because the amphiphilic block polymer containing the hydrophilic groups and the hydrophobic groups is able to self-aggregate into the micelle-like aggregates with a hydrophobic inner core and a hydrophilic outer shell in the aqueous solution, which can be applied to the medical field as a novel drug delivery system with broad prospects [3,4].

Alginate, as a natural anionic heteropolysaccharide mostly derived from brown seaweed, has been extensively studied and applied to many biomedical applications owing to its excellent advantages, such as low cost, non-toxicity, immunogenicity, and good biodegradation and biocompatibility [5,6,7]. In particular, alginate can perform mild gelation by divalent cations such as Ca^2+^, and the formed hydrogel can exhibit good coating, drug-loading, and sustained release properties, making it a good candidate as a drug carrier for biomedical application [8,9,10]. Alginate is a linear unbranched copolymer, consisting of two types of hexuronic acid residues: β-D-mannuronic acid (M) and α-L-guluronic acid (G) residues, which are (1,4) linked to each other by the glycosidic bonds and arranged in repeating GG (MM) blocks or alternating MG blocks [11,12]. Due to the abundant carboxyl and hydroxyl groups on the molecular chains, the raw alginate is very hydrophilic, which results in its poor compatibility with hydrophobic drug molecules, such as ibuprofen. [13,14]. Furthermore, the presence of these hydroxyl and carboxyl functional groups are very susceptible to forming intramolecular hydrogen bonds within the molecules, which gives rise to the highly stretched rigid structure of alginate chains that is not conducive to the loading of hydrophobic drugs [15]. In addition, the existence of numerous hydrophilic groups also makes alginate exhibit unpredictable and uncontrollable degradation kinetics and extensive water uptake properties, thus leading to its poor stability in biological buffers [16,17,18]. Therefore, with the raw alginate, it is difficult to achieve effective loading and controlled release of hydrophobic medicines in the biomedical field. Considering the abundant hydroxyl and carboxyl groups on the backbone of alginate, the chemical modification of alginate by hydrophobic groups may be an effective way to overcome its drawbacks as a hydrophobic pharmaceutical carrier because the chemical modification can be performed at the two secondary C–2 and C–3 hydroxyl groups and the C–6 carboxylic acid groups [19]. Furthermore, the grafting of hydrophobic segments onto the backbone of alginate can improve properties such as molecular flexibility, hydrophobicity, and physicochemical and biological characteristics, which make it capable of achieving the loading of hydrophobic drugs through its self-aggregation and prolonging its stability in biological medium [13,17].

**Scheme 1:**
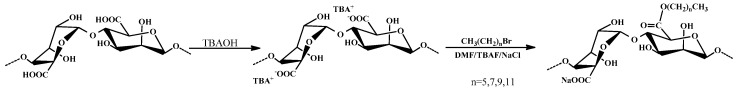
Synthetic routes of the neutralization of the alginic acid with TBAOH and the synthesis of AAD via the S_N_2 reaction.

Due to the multi-functional groups of alginate, a few research strategies or methods, involving the amidation, esterification, oxidation-reductive amination, and Ugi reaction (multicomponent condensation reaction named after Ivar Karl Ugi) on the chemical modification of alginate, have been reported [13,19]. For example, Yang et al. [20] prepared the amidic alginate derivatives by the amidation reaction for their application in the microencapsulation of λ-Cyhalothrin. Yang et al. [21] synthesized cholesterol grafted alginate derivatives using N, N’-bicyclohexyl carbodiimide as the coupling agent and 4- (N, N’-dimethylamino) pyridine as the catalyst. The fluorescence analysis showed that it could self-aggregate into micelles in a solution of 0.15 mol/L NaCl, with the critical aggregation concentration (CAC) of 0.33 g/L. Kang et al. [22] performed the oxidation-reductive amination reaction to synthesize a series of alginate-derived polymeric surfactants with the linear alkyl groups. The synthesized alginate-derived polymeric surfactants presented colloidally stable aggregates with a unimodal size distribution. Fang et al. [23] performed the Ugi reaction to prepare an amphiphilic alginate derivative, which was applied to stabilize Pickering emulsion for hydrophobic drug delivery. In general, the hydroxyl and carboxyl functional groups are very susceptible to forming intramolecular hydrogen bonds within the molecules, which gives rise to the fully stretched rigid skeleton structure of alginate chains and decreases the reactivity. Consequently, most chemical modifications require coupling agents and catalysts, including perchloric acid [24], 1-ethyl-3- (3-dimethylaminopropyl) carbodiimide hydrochloride (EDC·HCl) [20], 4- (N, N’-dimethylamino) pyridine [21], and 2-chloro-1-methylpyridinium iodide (CMPI) [18], to activate hydroxyl or carboxyl groups to improve the reactivity. However, a few previous works [25,26,27] have proposed a method for the chemical modification of alginate without the aid of a catalyst. This method mainly contains two processes: the neutralization of the alginic acid with tetrabutylammonium hydroxide (TBAOH) and its subsequent bimolecular substitution nucleophilic (S_N_2) reaction, illustrated in Scheme 1. Recently, Pawar et al. [28,29] improved this method with some modifications that increased the degree of substitution (DS) for alginate derivatives. They provided a strategy to dissolve tetrabutylammonium alginate (TBAA) in polar aprotic solvents with the presence of a dissolution promoter, such as tetrabutylammonium fluoride (TBAF), so that the esterification of TBAA could be achieved under homogeneous conditions via bimolecular substitution nucleophilic (S_N_2) reactions. The results showed that the resultant alginate ester derivatives could exhibit high chemical activity, with a DS close to 1.0. Although Pawar et al. [29] used TBAF-based two component solvent systems as media for the chemical modification of alginate via the S_N_2 reaction under homogeneous conditions, they only synthesized benzyl, butyl, ethyl, and methyl alginate esters as matrix polymers for the amorphous solid dispersion (ASD) of drugs. Research on the chemical modification of alginate using alkyl bromides with different lengths of carbon chain as the hydrophobic modifiers under homogeneous conditions via the S_N_2 reaction, especially the characterization, amphiphilic properties, self-aggregation behavior of the alginate ester derivatives, and the effects of the alkyl groups with different length of carbon chain (hexyl, octyl, decyl, and lauryl groups) on the physicochemical properties of the alginate ester derivatives, have been rarely reported so far. Furthermore, amphiphilic alginate ester derivatives could form micelle-like aggregates in aqueous media, so they could be widely used in encapsulating hydrophobic drugs for the development of hydrophobic pharmaceutical formulations for biomedical application.

In the current work, in order to extend the alginate applicability, thereby developing a promising biomedical material with great potential for the sustained release of hydrophobic medicine in drug delivery systems, the alkyl alginate ester derivative (AAD), including hexyl alginate ester derivative (HAD), octyl alginate ester derivative (OAD), decyl alginate ester derivative (DAD), and lauryl alginate ester derivative (LAD), were synthesized using alkyl bromides with different lengths of carbon chain as the hydrophobic modifiers under homogeneous conditions via the S_N_2 reaction. The structure and physicochemical properties of the resultant AAD were contrastively characterized by Fourier transform infrared spectroscopy (FT-IR), ^1^Hydrogen-nuclear magnetic resonance (^1^H NMR), X-ray diffraction (XRD), thermal gravimetric analysis (TGA), fluorescence spectrophotometer, transmission electron microscope (TEM), and dynamic light scattering (DLS). Moreover, the effects of the alkyl groups with different lengths of carbon chain (hexyl, octyl, decyl, and lauryl groups) on the physicochemical properties of AAD were examined. Finally, the loading and in vitro release of ibuprofen for the AAD microcapsules prepared by the emulsification method, and the cytotoxicity of AAD against the murine macrophage RAW264.7 cells, were also investigated.

## 2. Experimental Procedure

### 2.1. Materials

Alginic acid (90%, M_W_ = 137,100, M/G = 0.6), Tetrabutylammonium fluoride (TBAF, 99%), Tetrabutylammonium hydroxide (TBAOH, 25%), and ibuprofen (98%) were purchased from Aladdin Reagent Co., Ltd., Shanghai, China. 1-bromohexane (99%) and lauryl bromide (99%) were purchased from Adamas Reagent Co., Ltd., Shanghai, China. 1-bromooctane (98%) and 1-bromodecane (98%) were purchased from Alfa Aesar Chemical Co., Ltd., Shanghai, China. The murine macrophage RAW 264.7 cells were purchased from the Cell Bank of the Chinese Academy of Sciences, Shanghai, China. Dulbecco’s modified eagle medium (DMEM) was obtained from Gibco, Thermo Fisher Scientific, Waltham, MA, USA. Fetal bovine serum (FBS) was supplied by Biological Industries, Rehovot, Israel. A Cell Counting Kit-8 (CCK-8) was obtained from Dojindo Chemical Laboratories, Kumamoto, Japan. Other solvents, such as N, N-dimethylformamide (DMF), phosphate buffer saline (PBS), hydrochloric acid (HCl), sodium hydroxide (NaOH), sodium chloride (NaCl), ethanol, methanol, and ethyl acetate were also purchased from Aladdin Chemical Reagent Co., Ltd., Shanghai, China. These chemicals were analytical grade and used without further purification. All aqueous solutions were prepared with deionized water.

### 2.2. Homogeneous Synthesis of AAD with Different Hydrophobic Side Groups

As illustrated in Scheme 1, the synthesis route of AAD via the S_N_2 reaction involved the neutralization of the alginic acid with TBAOH to prepare tetrabutylammonium alginate (TBAA), followed by further esterification of the TBAA with alkyl bromides, which was based on the previous methods with some modifications [28,29]. Firstly, 8.0 g of alginic acid was dispersed in a 500 mL beaker containing 200 mL distilled water under stirring. Secondly, the aqueous TBAOH was added dropwise to completely dissolve the polymer until the pH was adjusted to 7~10, followed by suction filtration under vacuum using coarse filter paper to remove insoluble particulate impurities. In order to further remove the small unreacted substances, the resultant solution was dialyzed for 3 d and lyophilized to obtain pale yellow TBAA with a yield of 61.5%.

Subsequently, 1.2 g (2.59 mmol of hexuronic acid residues) of dry TBAA was fully dissolved in the 80 mL of DMF containing 0.8 g (3.06 mmol) of TBAF in a beaker under vigorous stirring. A certain amount of the alkyl bromide was then directly added to the blend solution, and the reaction was continuously stirred at ambient temperature for 24 h. Afterwards, 40 mL of 2.5 mol/L NaCl aqueous solution was added to the mixture, followed by stirring for another 2 h, so that the existing TBA^+^ ions could be replaced by Na^+^ ions. Finally, the resultant product was completely precipitated with a five times volume of ethyl acetate and separated by centrifuge at 8000 rpm to remove any residual reagents and byproducts. The product was dialyzed with deionized water for 3 d to remove residual impurities and then lyophilized to obtain the AAD. The experimental parameters for the chemical modification of alginate with different hydrophobic side groups are shown in Table 1. The AADs synthesized by 1-bromohexane, 1-bromooctane, 1-bromodecane, and lauryl bromide were labeled as HAD, OAD, DAD, and LAD, respectively. Meanwhile, sodium alginate (SA), prepared by neutralization of alginic acid with NaOH solution, was used as a reference.

### 2.3. Characterization of AAD

The DS values of the different AADs was calculated via saponification reaction according to the method in the literature [29]. The molecular structure and crystallinity of the AADs were characterized by Fourier transform infrared spectroscopy (FT-IR), ^1^Hydrogen-nuclear magnetic resonance (^1^H NMR) and X-ray diffraction (XRD). The FT-IR spectra of the samples were recorded on a Nicolet-6700 FT-IR spectrophotometer (Thermo Fisher Scientific, Waltham, MA, USA), and were acquired with 64 scans with a resolution of 4 cm^−1^ in the wavenumber range of 4000~400 cm^−1^. Approximately 2 mg of the sample was mixed with 100 mg of KBr, dried with an infrared lamp, and then compressed to semitransparent disks for spectroscopic analysis to determine the composition of the AAD functional groups. ^1^H NMR was performed on a Bruker AV 400 nuclear magnetic resonance spectrometer (Bruker, Fällanden, Switzerland) using a 5 mm NMR tube at 25 °C. The samples were dissolved in D_2_O (99%) to a concentration of approximately 10 mg/mL. XRD analysis of the samples was performed on a Bruker AXS/D8 advance diffract meter (Bruker, Cambridge, UK) using Cu-Kα (λ = 0.154 nm), and was operated at 40 kV and 100 mA in step scan mode at a scanning speed of 0.02°/s. X-ray diffraction measurements were performed over a 2θ range of 5°~60°. The thermal properties of AAD were measured by thermal gravimetric analysis (TGA), which was conducted using a TA Instrument Q600 Thermal Analyzer (TA Instrument, New Castle, DE, USA) in air at a heating rate of 20 K/min with the gas flow rate of 50 mL/min from 30 °C to 800 °C and cooling rate of 60 K/min.

### 2.4. Self-Aggregation Performance of AAD

The self-aggregation behavior of AAD was evaluated by fluorescence spectroscopy using pyrene as a fluorescence probe. The measurement was performed in 0.15 mol/L aqueous NaCl solution, which is beneficial to facilitate the aggregates of polymer samples. Fluorescence measurement was performed on a Hitachi F7000 fluorescence spectrophotometer (Hitachi, Honshu, Japan). Pyrene as a fluorescence probe was excited at 335 nm, and the emission spectrum was collected in the range of 350–500 nm at an integration time of 1s with a slit width of 2.5 nm. The morphology of the AAD micelle-like aggregates were observed with a JEM 2100 TEM (JEOL Co., Tokyo, Japan) at an acceleration voltage of 200 kV. Transmission electron microscope (TEM) images of the samples were obtained by placing a few drops of the aqueous dispersions of the samples onto a carbon-coated copper grid and evaporating the solvent prior to observation. The hydrodynamic diameter and zeta potential of AAD micelle-like aggregates were measured by DLS with a Malvern Nano-ZS90 Zetasizer (Malvern, Worcestershire, UK) at a scattering angle of 90° at 25 °C, employing an (He-Ne) argon laser (λ = 633 nm). The polymeric micelle-like aggregates solution was prepared at 60 °C and diluted to a concentration of 1.0 mg/mL to avoid multiple scattering.

### 2.5. Preparation of the Drug-Loaded AAD Microcapsules and Its Release Performance

The AAD microcapsules with the encapsulation of ibuprofen were prepared by the high-speed shearing method using AAD as the emulsifier. A certain amount of ibuprofen was dissolved in chloroform to prepare a chloroform solution with a drug concentration of 25 mg/mL. Then, 1 mL of this chloroform solution and 4 mL of 12.5 mg/mL AAD self-aggregated micelles solution were mixed well under high speed stirring to form the drug-loaded AAD microcapsule emulsion [17,20]. The microstructure of the drug-loaded microcapsules was observed by a fluorescent microscope (Nikon Ti-S FM, Tokyo, Japan). The microcapsules were stained with Nile red on the glass microscope slide, and they covered the coverslip for the observation. Additionally, the residual ibuprofen in the aqueous phase was extracted by chloroform, which was further measured by GC-MS (HP6890/5973MSD, Palo Alto, CA, USA) to determine the amount of residual ibuprofen. The encapsulation efficiency (EE) for the drug-loaded AAD microcapsules can be calculated using the following equation [30,31,32]:

(1) $$Encapsulation_{}efficiency_{}(EE)=\frac{total_{}ibuprofen-residual_{}ibuprofen}{total_{}ibuprofen}$$

The release of ibuprofen from the AAD microcapsules was performed in pH 7.4 phosphate buffered saline (PBS, 0.1 mol/L) medium at 37 °C. Approximately 2 mL of AAD microcapsules solution was placed into a dialysis bag with a molecular cut-off of 8000, and they were then immersed in 50 mL PBS in a centrifuge tube. An amount of 50 mL of the solution was replaced with the same volume of fresh PBS at different time intervals, thus avoiding the influences of the saturated solutions. The released ibuprofen for each time interval was measured by GC-MS, and the drug release procedure was performed in triplicate to calculate the standard deviation.

To further investigate its release mechanism, the Korsmeyer-Peppas model [33] was used to analyze the release profile.

(2) $$M_{t}/M_{\infty }=Kt^{n}$$

where *M_t_/M_∞_* is the fractional release of drug in time (*t*), *K* is a constant incorporating structural and geometrical characteristics of the delivery system, and *n* is the diffusion exponent characteristic of the release mechanism. For normal Fickian diffusion, the value of *n* = 0.5, and for case II diffusion, *n* = 1.0; the values of *n* intermediate between the above limits indicate non-Fickian transport [34].

### 2.6. Cytotoxicity of AAD

Cytotoxicity of the AAD was assessed using murine macrophage RAW264.7 cell, which were cultured with DMEM supplemented with 10% fetal bovine serum and 1% penicillin/streptomycin under the condition of 37 °C, 5% CO_2_, 95% air, and 100% relative humidity. The murine macrophage RAW264.7 cells were seeded at a density of 1.0 × 10^5^ cells/100 µL in 96-well plates with complete DMEM medium and cultured at 37 °C in a humidified incubator. Sterilized AAD was added at the final concentrations of 100, 200, 300, and 400 µg/mL. After 2 d of incubation, 10 µL of CCK-8 solution was added to each well. After 4 h of incubation, absorbance was measured at 450 nm using an ELISA plate reader and then converted into a macrophage cell viability as follows:

(3) $$\mathrm{Cell}\;\mathrm{viability}=\left[\frac{A_{s}-A_{b}}{A_{c}-A_{b}}\right]\times 100\%\;$$

where A_s_ is the absorbance of the sample group, Ac is the absorbance of the control group, and A_b_ is the absorbance of the blank group. Simultaneously, the cells cultured on the 96-well plates without AAD were served as the blank control group.

## 3. Results and Discussion

### 3.1. Synthesis and Characterization of AAD

**Figure 1:**
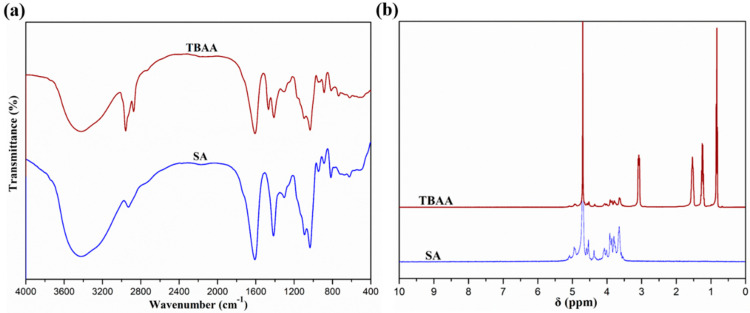
(**a**) FT-IR and (**b**) ^1^H NMR spectra of SA and TBAA.

According to previous work [28,29], we attempted to conduct the homogeneous synthesis of AAD in *N*,*N*-dimethylformamide/tetrabutylammonium fluoride (DMF/TBAF), and partially esterified hexyl, octyl, decyl, and lauryl alginates were synthesized via the S_N_2 reaction with the corresponding alkyl halides. As shown in Scheme 1, the homogeneous synthesis of AAD in DMF/TBAF consisted of two processes: (1) synthesis of TBAA intermediates; (2) esterification of TBAA intermediates. The S_N_2 reaction could be carried out in a homogeneous solution because TBAA could be completely dissolved in DMF with the aid of 1% TBAF. The successful synthesis of TBAA intermediates could be verified by FT-IR spectra and ^1^H NMR spectra, as shown in Figure 1.

It was observed from Figure 1a that the SA displayed the main characteristic bands at 2929, 1610, and 1414 cm^−1^, which were respectively assigned to the C–H stretching vibration of the polysaccharide structure and the asymmetric and symmetric stretching vibration of –COO– [35]. The absorption bands at 1094, 1034, and 951 cm^−1^ were attributed to C–O and C–O–C stretching vibration on the polysaccharide skeleton, respectively [36]. In comparison with SA, the TBAA showed the chemical structure difference in its FT-IR spectrum, and new weak bands at 2874 and 1474 cm^−1^, owing to the –CH_2_– stretching vibration and –CH_3_ bending vibration of TBA^+^, could be observed. These results indicated the successful synthesis of TBAA intermediates through the neutralization of the alginic acid with TBAOH. In addition, it can be seen from Figure 1b that the signal peaks at 5.5~3.0 ppm were ascribed to the proton signal peaks on the alginate backbone of SA [20]. In comparison with SA, the TBAA showed new proton signal peaks at 3.10~3.00, 1.42~1.66, 1.25~1.20, and 0.86~0.80 ppm, which were classified as –CH_2_– and –CH_3_ of TBA^+^. This result further proved the successful synthesis of TBAA intermediates.

To investigate the effects of alkyl groups with different lengths of carbon chain (hexyl, octyl, decyl, and lauryl groups) on the physicochemical properties of AAD, the HAD, OAD, DAD, and LAD were synthesized using the corresponding alkyl halides with the N_Alkyl bromide_/N_Hexuronic_ molar ratios fixed at 0.5 via the S_N_2 reaction. The reaction parameters for the synthesis of AAD with different DS and yields are presented in Table 1. It can be observed that the DS of HAD, OAD, DAD, and LAD was close to 40%. Meanwhile, to prepare different DS of HAD, various N_1-Bromohexane_/N_Hexuronic_ molar ratios were performed to achieve the chemical modification. The different DS and yields of HAD with the N_1-Bromohexane_/N_Hexuronic_ values of 0.3, 0.5, and 1.2 are also presented in Table 1. It can be seen that the DS and yield of HAD increased with the increase of N_1-Bromohexane_/N_Hexuronic_ values. Even when the N_1-Bromohexane_/N_Hexuronic_ molar ratio was 1.2, the DS of HAD reached approximately 100%. This result directly indicated that the S_N_2 reaction for the chemical modification of SA was active. To note, it was reported that the AAD grafted with the alkyl chains was able to associate to form self-aggregated micelles through both intra- and intermolecular hydrophobic interactions [21]. However, these properties were obtained only within a narrow range of DS. At low DS, AAD was not enough to drive its self-aggregation, while at high DS, the polymer derivatives were no longer soluble in water [37]. For this reason, we selected the AAD with the DS close to 40% to develop the hydrophobic pharmaceutical formulations.

**Figure 2:**
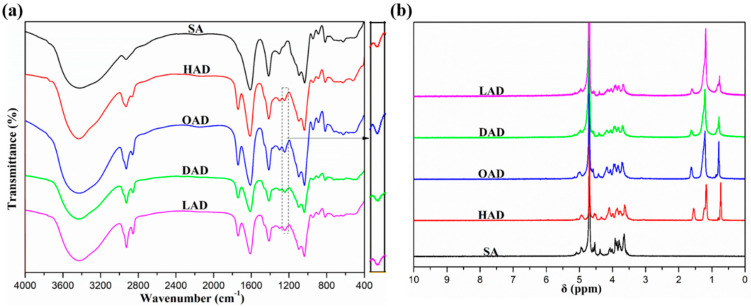
(**a**) FT-IR and (**b**) ^1^H NMR spectra of SA, HAD, OAD, DAD and LAD.

Figure 2a represents the FT-IR spectra of SA, HAD, OAD, DAD, and LAD. Compared with the spectrum of SA, HAD, OAD, DAD, and LAD exhibited the additional weak bands in addition to the basic characteris37tic absorption bands of SA. The absorption bands of HAD, OAD, DAD, and LAD at 2930, 2928, 2927, and 2926 cm^−1^ were significantly enhanced, and were ascribed, respectively, to the stretching vibration of –CH_3_ on the hexyl, octyl, decyl, and lauryl groups. Furthermore, the additional bands appearing at 2860, 2860, 2858, and 2926 cm^−1^ were assigned, respectively, to the –CH_2_– stretching vibration of the hexyl, octyl, decyl, and lauryl groups. In addition, the additional bands appearing at 1741, 1741, 1741, and 1740 cm^−1^ were owing to stretching vibration of C=O on the ester group, while the other new bands appearing at 1244, 1245, 1243, and 1244 cm^−1^ were attributed to the stretching vibration of C–O on the ester group [29]. These results indicated that the hexyl, octyl, decyl, and lauryl groups had successfully grafted onto alginate via the S_N_2 reaction to generate the AAD.

Figure 2b shows the ^1^H NMR spectra of SA, HAD, OAD, DAD, and LAD. It can be observed that all the samples exhibited proton peaks ranging from 5.0 to 3.5 ppm, which were assigned to the proton signal of native alginate backbone [20]. However, in comparison with SA, the spectra of HAD, OAD, DAD, and LAD showed new proton peaks at 1.70~1.60, 1.40~1.20, and 0.90~0.70 ppm, which were attributed to –CH_2_– and –CH_3_ signals on the hexyl, octyl, decyl, and lauryl groups, and these new peaks gradually increased with the increase of the carbon chain length of the alkyl groups [7,20,35]. The results further proved that the hydrophobic alkyl groups were successfully grafted onto alginate molecular chains.

**Figure 3:**
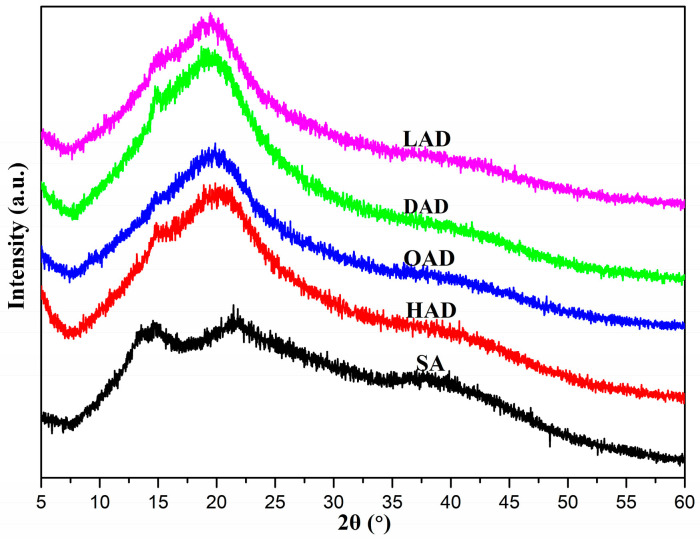
X-ray diffractograms of SA, HAD, OAD, DAD, and LAD.

XRD is the most direct and effective method to analyze the change of crystal structures of alginate in the S_N_2 reaction. It can be observed from Figure 3 that SA, HAD, OAD, DAD, and LAD exhibited weak crystalline diffraction peaks, indicating their amorphous structures. The diffraction peaks of SA at around 15° and 22° were typical characteristic peaks of the hydrated crystalline structures resulting from the intramolecular hydrogen bonds of SA [38,39], but HAD, OAD, DAD, and LAD revealed a sharp diffraction peak at 2θ = 14.8° and a wider diffraction peak at 2θ = 20° after the chemical modification, which was similar to the crystal structure characteristics of alginate derivatives reported by Chen et al. [35]. By contrast, the diffraction peak of alginate decreased and became broad after the esterification modification, which showed that the microcrystalline structures of AAD had changed; therefore, the transformation of these crystal diffraction peaks for SA indirectly indicated that the esterification modification had weakened and destroyed the intramolecular hydrogen bonds of alginate, thus enhancing its molecular flexibility [7].

**Figure 4:**
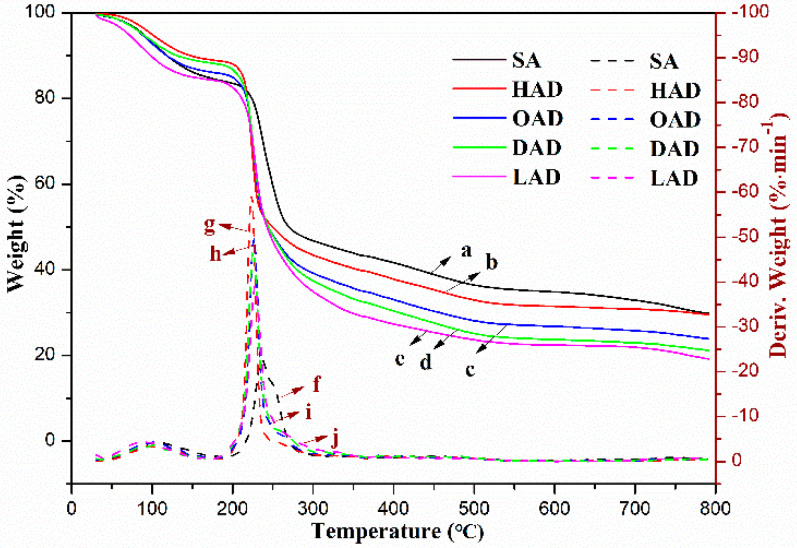
Thermogravimetric analysis of (**a**) SA, (**b**) HAD, (**c**) OAD, (**d**) DAD, and (**e**) LAD as well as derivative thermograms (DTG) of (**f**) SA, (**g**) HAD, (**h**) OAD, (**i**) DAD, and (**j**) LAD.

Thermogravimetric analysis was utilized to estimate the different thermal stabilities of SA and HMAD, which could indirectly reflect the change of molecular structures of alginate in the S_N_2 reaction. It can be observed from Figure 4 that the weight losses of SA, HAD, OAD, DAD, and LAD at 800 °C were 70.2%, 70.5%, 76.1%, 78.9%, and 80.9%, respectively. Moreover, SA, HAD, OAD, DAD, and LAD displayed two main weight loss stages from their DTG curves: one was attributed to the losses of physically adsorbed water at low temperature (80~120 °C), the other was ascribed to the decomposition of polymer molecular at high temperature (200~250 °C) [40]. It can be observed that, with the increase of the carbon chain lengths of the hydrophobic side groups, the hydrophobicity of AAD is gradually enhanced, which lead to the reduction of the physically adsorbed water, thus decreasing the weight loss in the temperature range of 50~200 °C. When the temperature was gradually rising, the molecular chain of the polymer broke down and generated CO, CO_2_, and H_2_O [30,31], resulting in the rapid decrease of weight. The initiating decomposition temperatures of SA, HAD, OAD, DAD, and LAD were 237, 224, 226, 223, and 222 °C, respectively. Therefore, it was obvious that the AAD had lower thermal stability due to the decrease of the carboxyl groups of the polymer and the formation of ester bonds that destroyed the polymeric intramolecular hydrogen bond. These results further imply the esterification of alginate via the S_N_2 reaction was successful.

### 3.2. Colloidal Interface Activity of AAD

**Figure 5:**
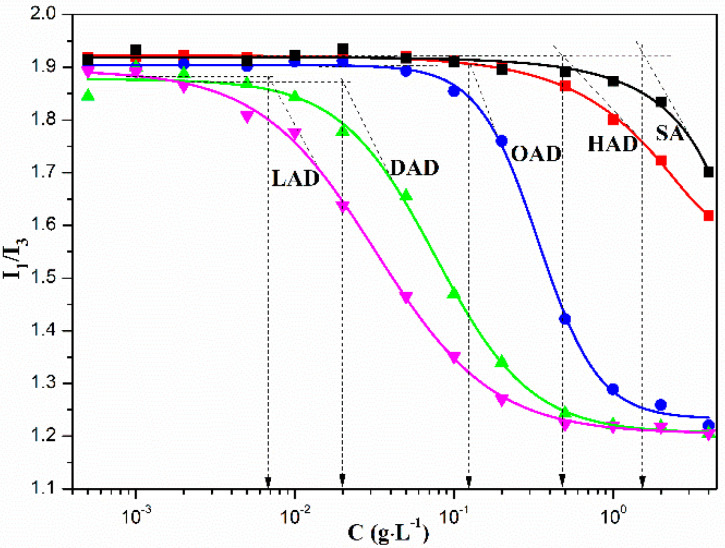
Plots of pyrene fluorescence intensity I_1_/I_3_ vs. the concentration of SA, HAD, OAD, DAD, and LAD in aqueous NaCl solution.

The colloidal interface activity of AAD was evaluated by fluorescence spectroscopy, transmission electron microscopy (TEM), and dynamic light scattering (DLS). The photophysical characteristic of pyrene depended on its surrounding hydrophilic and hydrophobic environments, which could be applied to detect the self-aggregation behavior of AAD [41,42]. In the present work, the amphiphilic property of AABD and its critical aggregation concentration (CAC) value were determined by the pyrene fluorescence probe technique. Pyrene has five peaks in the emission spectra, and the ratio value of the fluorescence intensity of the first peak at 372 nm (I_1_) to the third peak at 383 nm (I_3_) was influenced by the micro-environmental polarity in the aqueous solution [7,21]. Pyrene, commonly used as the fluorescent probe, is insoluble in the aqueous medium, but it can be encapsulated in the hydrophobic micro-domains formed by the alkyl chains of AAD [7]. It can be accepted that the intensity ratio values of I_1_ and I_3_ (I_1_/I_3_) are related to the micro-environmental polarity surrounding pyrene molecules, which decreases with the reduction of the micro-environmental polarity. When the hydrophobic alkyl groups, such as hexyl, octyl, decyl, and lauryl groups, drove AAD to form self-aggregated micelle, the ratio value of I_1_/I_3_ significantly decreased, so the lower the ratio of I_1_/I_3_, the higher of the hydrophobicity for AAD [21]. As shown in Figure 5, the ratio of I_1_/I_3_ for AAD was significantly lower than that of SA at the same concentration, indicating that the AAD emerged as having the higher hydrophobicity. Furthermore, the ratio of I_1_/I_3_ of AAD gradually decreased with the increase of its concentration. The decrease in the ratio of I_1_/I_3_ can be applied to evidence hydrophobic micro-domain formation due to intermolecular hydrophobic associations after the formation of self-aggregated micelle. Moreover, the critical aggregation concentration (CAC) of AAD, determined from the change of fluorescence intensity ratio (I_1_/I_3_) in polymer concentration, is the lowest concentration of self-aggregation formation by intramolecular or intermolecular associations [4]. The CAC value of SA, HAD, OAD, DAD, and LAD were 1.55, 0.48, 0.12, 0.02, and 0.0068 g/L, respectively, which indicated that AAD possessed both hydrophilic and hydrophobic properties. In particular, the OAD, DAD, and LAD curves of I_1_/I_3_ vs. the concentration were consistent with the results reported by Yan et al. [7], which revealed a typical characteristic with two plateaus, at low concentrations and at high concentrations, respectively, similar to the classic surfactant. Furthermore, it was apparent that the CAC of AAD was significantly lower than that of SA. The lower the CAC value, the higher the stability of the micelles at low concentrations in aqueous medium or blood circulation systems post administration. The results implied that the S_N_2 reaction was an effective approach to endow the AAD with good amphiphilic functionality.

**Figure 6:**
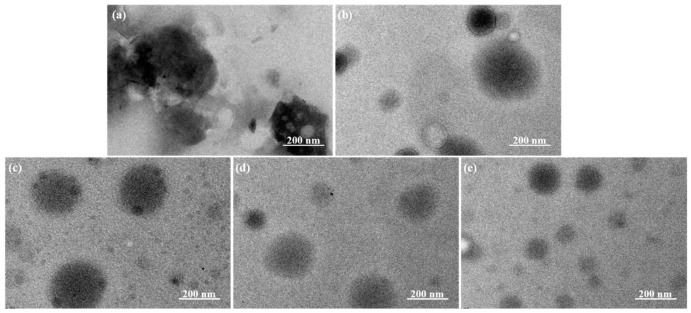
TEM images of (**a**) SA aggregates, (**b**) HAD, (**c**) OAD, (**d**) DAD, and (**e**) LAD self-aggregated micelles.

Intermolecular hydrophobic associations of amphiphilic AAD resulted in the formation of self-aggregated micelle at a concentration higher than its CAC in 0.15 mol/L aqueous NaCl solution. The morphology of HAD, OAD, DAD, and LAD self-aggregated micelles could be measured by TEM observation. As shown in Figure 6, SA only generated the irregular self-aggregates because its molecular chain contains a large number of hydroxyl groups and negatively charged carboxyl groups, and it is easy to form strong intramolecular hydrogen bonds and electrostatic repulsion in 0.15 mol/L aqueous NaCl solution [21]. However, AAD could form regular self-aggregated micelles with a spherical shape and good structural integrity through molecular self-assembly. This result was ascribed to the hydrophobic associations and hydrogen bonding of the hexyl, octyl, decyl, and lauryl groups that were advantageous in terms of the self-aggregation behavior of amphiphilic AAD, which not only facilitated the stability of self-aggregated micelle but also influenced the morphology of the micelles [43,44].

**Figure 7:**
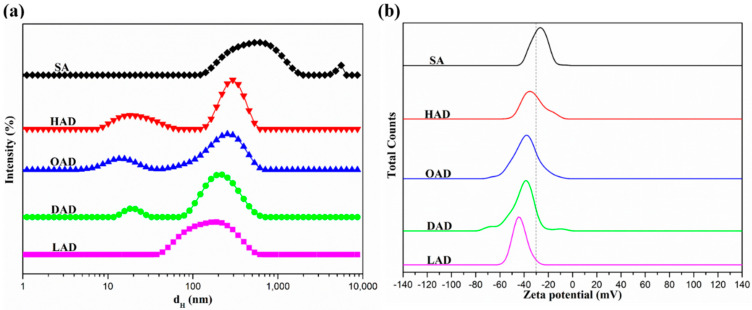
(**a**) Hydrodynamic diameter distribution and (**b**) Zeta potential distribution of SA, HAD, OAD, DAD, and LAD self-aggregated micelles.

The colloidal property of AAD self-aggregated micelles was further investigated by dynamic light scattering (DLS). As shown in Figure 7a, the AAD self-aggregated micelle exhibited a narrow hydrodynamic diameter (d_H_) distribution in comparison with the SA aggregates. The average d_H_ of SA, HAD, OAD, DAD, and LAD self-aggregated micelles were 570.2 nm (PDI = 0.49), 285.3 nm (PDI = 0.29), 245.0 nm (PDI = 0.36), 210.2 nm (PDI = 0.32), and 180.5 nm (PDI = 0.21), respectively. As the alkane chain increased, the average d_H_ gradually decreased. The growth of the carbon chain length of the hydrophobic side groups enhanced the attraction between the ends of the tail, which caused strong hydrophobic associations, resulting in the reduction of d_H_. These results were attributed to the rigid and extended chain characteristics of SA molecules in aqueous media that led to bigger diameters for the SA aggregates [45], while the grafting of the hydrophobic side groups of the hexyl, octyl, decyl, and lauryl groups broke the intramolecular hydrogen bonds and enhanced the molecular flexibility that enabled itself to curl freely to form smaller diameters for the AAD self-aggregated micelle [35,45]. Moreover, from Figure 7b, due to the enhancement of molecular flexibility, which exposed more residual free-curling carboxyl groups on the surface, the SA, HAD, OAD, DAD, and LAD self-aggregated micelles revealed the relatively high negative zeta potential at −27.4, −44.8, −39.2, −38.3, and −34.4 mV, respectively, as a result of the presence of the negatively charged carboxyl groups [30]. The zeta potential of the AAD self-aggregated micelle was significantly lower than that of the SA aggregates, which was attributed to the fact that the AAD with both the hydrophilic main chains and hydrophobic side groups was able to self-aggregate into core-shell architectures where the hydrophilic carboxyl groups were exposed, thus reducing its zeta potential. It was reported that the self-aggregated micelle was stable in aqueous solution when its zeta potential was lower than −30 mV [7,46]. Therefore, the HAD, OAD, DAD, and LAD self-aggregated micelle could be significantly stable in aqueous solution due to the strong electrostatic repulsion that can prevent aggregation among the micelles.

Table 2 shows the comparison results of HAD, OAD, DAD, and LAD, including the DS and CAC value and average d_H_ and zeta potential. It was found that, with the increase of carbon chain length of the hydrophobic side groups, the DS and CAC value of AAD were gradually reduced, indicating that the chemical modification of alginate with the shorter alkyl groups revealed the higher reaction activity, and the longer the hydrophobic side groups of AAD, the stronger the hydrophobic association, thus displaying better amphiphilic properties [7,20]. In addition, the average d_H_ and zeta potential of AAD also decreased with the increase of the carbon chain length of the hydrophobic alkyl groups. This result shows that the longer the hydrophobic side groups of AAD, the stronger the hydrophobic association, making the AAD more prone to self-aggregation, thereby exhibiting higher colloidal interface activity, which was suitable to develop the hydrophobic pharmaceutical formulations [21].

### 3.3. Drug-Loading and Release Performance of AAD Microcapsules

**Figure 8:**
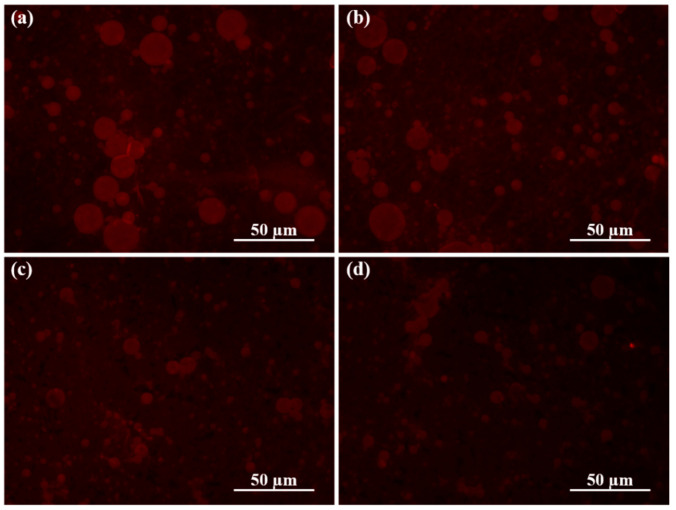
Fluorescent images of the dyed microcapsules emulsions stabilized by (**a**) HAD, (**b**) OAD, (**c**) DAD, and (**d**) LAD under dark field.

Thanks to the good colloidal interface activity of AAD, it could be used to prepare drug-loaded microcapsules by the emulsification method. The microstructures of the fabricated drug-loaded AAD microcapsules were observed by fluorescent microscope after fluorescence staining, and they are presented in Figure 8. It can be observed that the emulsion droplets were in the regular spherical shape, indicating that HAD, OAD, DAD, and LAD possessed emulsifying properties similar to traditional surfactants. The emulsion droplets appeared red in the dark field for the Nile red excitation, which indicates that the drug-loaded AAD microcapsule emulsion was an oil-in-water emulsion type because the oil-soluble Nile red only existed in the oil phase and appeared red for its excitation [47]. According to Equation (1), the encapsulation efficiency (EE) of HAD, OAD, DAD, and LAD drug-loaded microcapsules was calculated to be 71.6%, 75.3%, 77.1%, and 78.8% (Table 3) which were much higher than that of SA drug-loaded microcapsules. Moreover, with the increase of the carbon chain length of the hydrophobic side groups, the EE of AAD gradually increased for the enhancement of the hydrophobic associations. This result indicated that the modification reaction had greatly improved the loading performance of the AAD for hydrophobic drugs [17,35].

**Figure 9:**
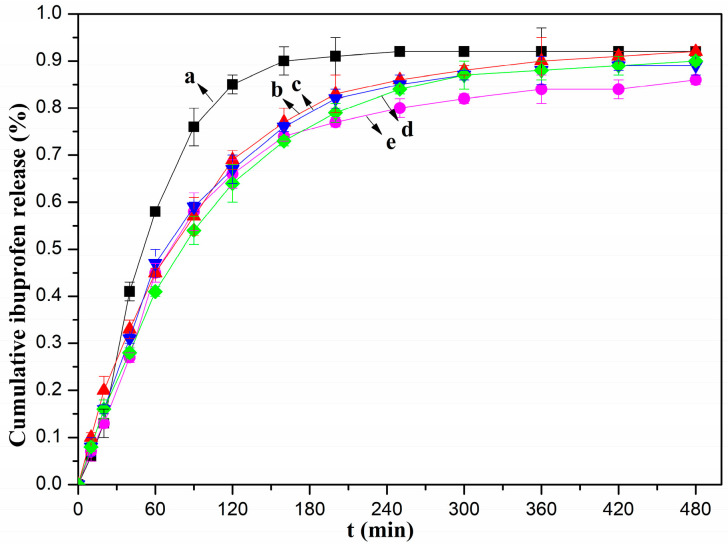
Release of ibuprofen from (**a**) SA, (**b**) HAD, (**c**) OAD, (**d**) DAD, and (**e**) LAD microcapsules (error bars represent the standard deviation of three replicates).

Ibuprofen is a commonly used non-steroidal anti-inflammatory drug that has been widely applied to the treatment of various musculoskeletal diseases and pain symptoms. The release of ibuprofen from the drug-loaded microcapsules was achieved through self-diffusion of the drug and the swelling and degradation of the drug-loaded microcapsules [31]. Figure 9 shows the release profiles of ibuprofen from SA, HAD, OAD, DAD, and LAD microcapsules in pH 7.4 PBS at 37 °C. It can be observed that the SA microcapsules revealed a significant burst release, and approximately 85% of the drug was quickly released within the first 120 min. However, at the same time, the release rate of ibuprofen in the AAD microcapsules was significantly lower than that of SA microcapsules, and it could be released continuously within 480 min, indicating that the hydrophobic inner cavity of AAD microcapsules can effectively solubilize hydrophobic ibuprofen, thus slowing down the diffusion rate of the drug and reducing the drug release rate. Meanwhile, the increase of the carbon chain length of the hydrophobic side groups for AAD was more conducive to achieving the controlled release performance because the enhancement of the hydrophobic associations could effectively retarded the drug release rate. The prolongation of release time may be helpful to improve the drug efficacy and drug utilization in actual drug therapy [48,49]. Through the Peppas model fitting in Table 3, the model correlation coefficients R^2^ of SA and AAD microcapsules were higher than 0.99, indicating that the release curves were well fitted by the model equation. Additionally, the characteristic indexes, n, of the release process of the SA, HAD, OAD, DAD, and LAD microcapsules were 0.9310, 0.8524, 0.8603, 0.7990 and 0.8165, respectively, indicating that the release processes of the SA and AAD microcapsules belonged to the non-Fickian diffusion model, indicating that the swelling and degradation of the AAD microcapsules was related to the diffusion of the loaded drug, which jointly controlled the release rate of the ibuprofen [30].

### 3.4. Cytocompatibility of AAD

**Figure 10:**
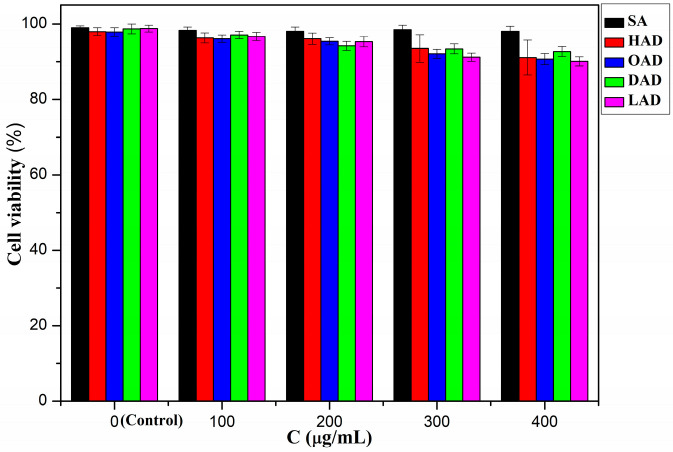
CCK-8 assay for cytotoxicity of RAW 264.7 cells cultured on SA, HAD, OAD, DAD, and LAD after 2 days incubation.

The cytotoxicity of the HAD, OAD, DAD, and LAD was evaluated on the murine macrophage RAW264.7 cell by CCK-8 assay. As shown in Figure 10, the murine macrophage RAW264.7 cell with various concentration of AAD microcapsules displayed a similar cell viability to the SA, which was close to the control, indicating that the RAW264.7 cell could be viable and proliferate well on the HAD, OAD, DAD, and LAD [50]. However, the cell viability gradually decreased with the increase of the AAD concentration, which implied that excessively high concentrations of AAD might inhibit the viability of cells. However, when the concentration of AAD was as high as 400 μg/mL, the cell viability still remained above 90% after 2 days of incubation, exhibiting good cytocompatibility of AAD. Therefore, in view of the good hydrophobic drug-loading capacity, release performance, and cytocompatibility of AAD, they can be applied to the pharmaceutical field for medical applications.

## 4. Conclusions

In summary, we attempted to conduct the S_N_2 reaction to synthesize AAD, including HAD, OAD, DAD, and LAD using alkyl bromides with different lengths of carbon chain as the hydrophobic modifiers under homogeneous conditions. Experimental results showed that HAD, OAD, DAD, and LAD was successfully synthesized, and the grafting of the hydrophobic alkyl groups onto the alginate molecular backbone had weakened and destroyed the intramolecular hydrogen bonds, thus enhancing the molecular flexibility of the alginate. Therefore, the resultant AAD could exhibit a good amphiphilic property to self-aggregate into spherical micelles with an average hydrodynamic diameter of 285.3~180.5 nm and zeta potential of approximately −44.8~−34.4 mV due to the intra- or intermolecular hydrophobic associations. With the increase of the carbon chain length of the hydrophobic side groups, the EE of AAD gradually increased for the enhancement of the hydrophobic associations, and the increase of the carbon chain length of the hydrophobic side groups for AAD was more conducive to achieving the controlled release performance for the enhancement of the hydrophobic associations that effectively retarded the drug release rate. The swelling and degradation of AAD microcapsules and the diffusion of the loaded drug jointly controlled the release rate of ibuprofen. Furthermore, the cytotoxicity test results showed that the AAD also displayed low cytotoxicity to the murine macrophage RAW264.7 cells. Therefore, the synthesized AAD with good amphiphilic property, colloidal interface activity, drug-loading performance, and cytocompatibility exhibited a great potential for the development of hydrophobic pharmaceutical formulations for biomedical application.

## Figures and Tables

**Table 1:** Reaction parameters for the synthesis of AAD with different DS and yield.

Scheme	N_Alkyl bromide_/N_Hexuronic_	TBAA (mmol)	Alkyl Bromide (mmol)	DS (%)	Yield (%) ^a^
HAD	0.3	2.59	0.78	26.2	76.8
HAD	0.5	2.59	1.30	44.6	79.5
HAD	1.2	2.59	3.11	100.0	83.4
OAD	0.5	2.59	1.30	42.3	78.4
DAD	0.5	2.59	1.30	41.5	79.7
LAD	0.5	2.59	1.30	40.1	78.6

^a^ Yield = actual yield/theoretical yield × 100%.

**Table 2:** Comparison of performance parameters of AAD with different hydrophobic side groups.

Sample	DS (%)	CAC Value (g/L)	Average d_H_ (nm)	Zeta Potential (mV)
HAD	44.6	0.48	285.3	−34.4
OAD	42.3	0.12	245.0	−38.3
DAD	41.5	0.02	210.2	−39.2
LAD	40.1	0.0068	180.5	−44.8

**Table 3:** SA, HAD, OAD, DAD, and LAD microcapsules fitting results for the ibuprofen release data.

Formulation Code	EE	n	R^2^	Diffusion Mechanism
SA microcapsules	25.5% ± 3.2%	0.9310 ± 0.0064	0.9815	non-Fickian
HAD microcapsules	71.6% ± 2.8%	0.8524 ± 0.0061	0.9925	non-Fickian
OAD microcapsules	75.3% ± 3.0%	0.8603 ± 0.0058	0.9930	non-Fickian
DAD microcapsules	77.1% ± 2.3%	0.7990 ± 0.0057	0.9911	non-Fickian
LAD microcapsules	78.8% ± 2.5%	0.8165 ± 0.0050	0.9904	non-Fickian

## Data Availability

The data presented in this study are available on request from the corresponding author.
